# Double isolated asynchronous duodenal perforation due to abdominal blunt trauma in a child: A case report

**DOI:** 10.1016/j.ijscr.2020.09.183

**Published:** 2020-10-02

**Authors:** V. Briganti, S. Tursini, S. Ianniello, A. Cortese, R. Faggiani

**Affiliations:** a Pediatric Surgery Operative Unit, San Camillo, Forlanini Hospital, Rome, Italy; b Emergency Radiology Operative Unit, San Camillo, Forlanini Hospital, Rome, Italy; c Radiology Operative Unit Operative Unit, San Camillo, Forlanini Hospital, Rome, Italy; d Gastroenterology and Diagnostic and Operative Digestive Endoscopy Operative Unit, San Camillo, Forlanini Hospital, Rome, Italy

**Keywords:** Duodenal perforation, Blunt abdominal trauma, Children

## Abstract

**Background:**

Isolated duodenal perforation following blunt abdominal trauma is a rare injury in children. Bicycle accidents (falling on to the handlebar) are a frequent cause of blunt abdominal trauma in children and may occasionally be associated with isolated duodenal perforation (IDP). Prompt diagnosis and surgical treatment are vital to prevent increased morbidity and mortality.

**Case presentation:**

We report the rare case of an 11-year-old boy admitted for blunt abdominal trauma and treated for an asynchronous double IDP. The first perforation, located on the 2nd/3rd portion of the duodenum, was promptly diagnosed by contrast-enhanced abdominal CT scan after a negative US scan, five hours after injury, and the lesion repaired with a single stitch suture. The second duodenal perforation appeared in the duodenal bulb as a worsening biliary leakage, 48 h after the primary suture of the initial lesion. The perforation was initially seen by digestive endoscopy and sutured in the same way as the first lesion. A third laparotomy was needed 4 days later due to an intestinal obstruction, after which the patient was recovered completely and was discharged home.

**Discussion and conclusion:**

IDP is a rare consequence of blunt abdominal trauma, and is normally associated with a lesion of other organs, such as the pancreas or bile duct. A delayed diagnosis strongly increases the incidence of morbidity and mortality, and different kinds of surgical management have been proposed, depending on the type of lesion. To our knowledge, this is the first case described in literature of a double isolated asynchronous duodenal perforation following blunt abdominal trauma in children.

## Highlights

• This is the first case described in literature of a double asynchronous isolated perforation of the duodenum.
• Timing of diagnosis and treatment are described.
• Radiologic findings are provided.

## 1 Introduction

This case has been reported in line with the SCARE criteria [1].

Blunt abdominal trauma is most frequent in the paediatric population, and isolated duodenal lesions are infrequent, accounting for just 3–5 % of all trauma cases [2] due to the position of the duodenum, partially protected by the retroperitoneum. This position, close to the vertebral column, can lead to blowout lesions, while the close proximity of the duodenum to many other vital structures means that lesions to this part of the bowel are normally associated with injuries to other organs [3]. This particular anatomical characteristic, together with the rarity of IDP and its subtle clinical features, makes the diagnosis and management of such lesions potentially challenging. We describe the case and the management of an 11-year-old boy admitted for blunt abdominal trauma with a double asynchronous duodenal perforation in two different locations of the organ, not previously reported in literature.

## 2 Case report

**Fig. 1:**
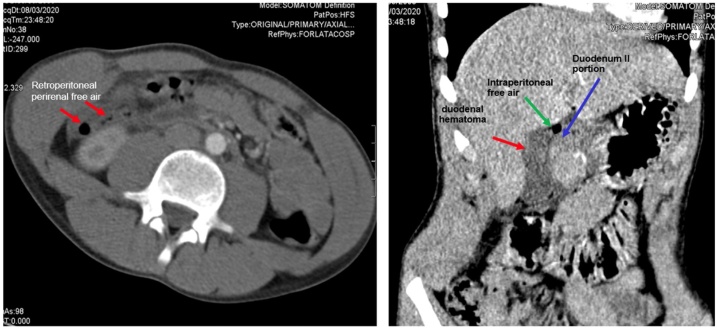
Abdominal CT scan showing retroperitoneal (a) and intrabdominal free air and a duodenal haematoma (b).

**Fig. 2:**
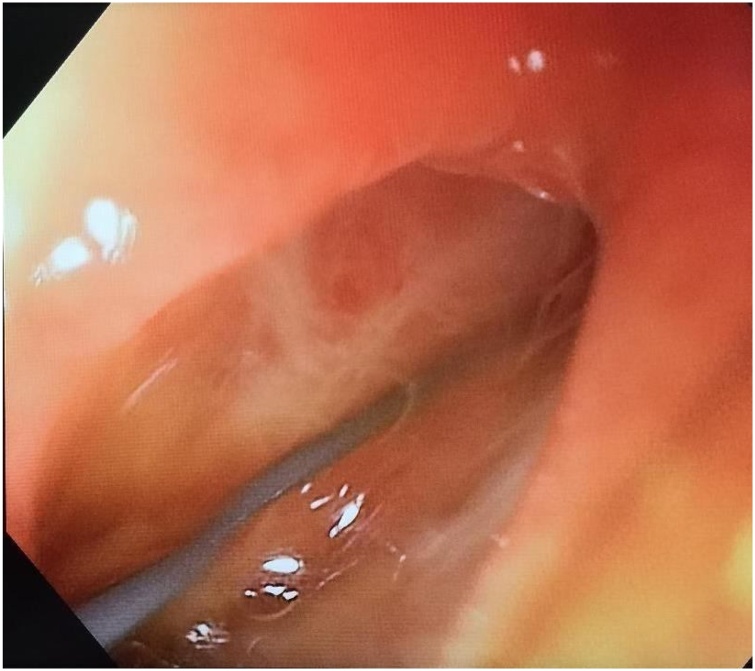
Duodenal bulb perforation with guidewire.

**Fig. 3:**
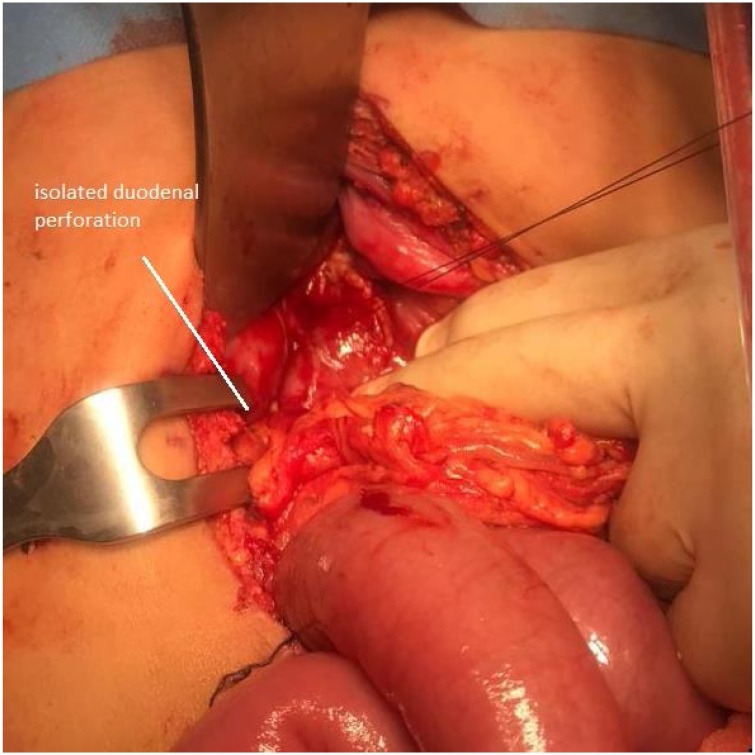
The second duodenal perforation in the antrum.

An 11-year-old boy was admitted to a suburban general hospital due to blunt abdominal trauma (caused by a bicycle handlebar) and was transferred to our department five hours after the initial injury due to worsening abdominal pain. On arrival, the patient showed right upper quadrant and epigastric abdominal pain with cutaneous swelling, and was alert and afebrile, with a pulse rate of 110/min. Laboratory tests showed an increased WBC count (20.35 × 10 e3/uL - N-91 % with a reference range of 4.0–10.0 e3/uL). Abdominal US was negative, but due to his worsening overall condition, the patient underwent a contrast-enhanced CT scan that showed a suspected duodenal perforation in the II portion of the duodenum, with free air in the retroperitoneum and free fluid in the peritoneal space, wall thickening, and duodenal haematoma (Fig. 1a and b). The patient immediately underwent an exploratory laparotomy that showed a diffuse haematoma of the second portion of the duodenum, with a laceration of the peritoneal attachment and a perforation of the anterior surface of the duodenum near the junction of the 2nd and 3rd duodenal tract, which opened into the peritoneum with biliary leakage (II type laceration – AAST-OIS) [4]. Following a careful Kocker manoeuvre to examine the entire 2nd and 3rd portion of the duodenum, the perforation was repaired with a single stitch suture and omental patch; duodenal bulb, biliary tree and pancreas were intact. Two drains were left in the peritoneum, the first close to the perforation and the second in the pelvis. A percutaneous central catheter was also inserted through the right internal jugular vein. The patient was admitted to the ward in good condition and with TPN. 48 h later, biliary fluid began to appear in the drain, with no abdominal sign of peritonitis, nor worsening of the patient’s overall condition. A new contrast-enhanced CT scan showed a small amount leakage in the I-II portion of the duodenum, and conservative treatment was started. The amount of fluid increased over the following 24 h, reaching a volume of around 800 mL/day. On the suspicion of a failure of the previous suture, the patient underwent an endoscopy to insert a biliary drain. The test showed a new perforation in the duodenal bulb, and a guidewire was introduced (Fig. 2). The subsequent laparotomy showed a single perforation on the anterior wall of the duodenal bulb (II type laceration – AAST-OIS), immediately after the pylorus (Fig. 3), while the previous perforation was perfectly closed, as seen at the previous endoscopy. The perforation was repaired with a single stitch suture and omental patch, and a new drain was left in place. After 48 h in ICU, the patient was readmitted to the ward in good condition and on TPN. After three days without any biliary leakage from the drain, the patient began presenting with biliary vomiting. X-ray showed an intestinal obstruction, prompting a new laparotomy which showed an ileal obstruction due to peritoneal adhesion. The post-operative period was uneventful, and the drain was removed on the 5th post-operative day. He was gradually returned to oral feeding and was discharged on the 10th post-operative day in a good condition. At three months’ follow up, the patient is completely recovered.

## 3 Discussion

Injuries to the duodenum are uncommon due to its retroperitoneal position, and the cause is rarely “blunt” trauma [5]. The duodenum is almost completely retroperitoneal, except for the anterior wall of the first part, which is mobile and intraperitoneal. The duodenum lies in strict proximity to other organs (pancreas, bile duct) and major vessels, meaning that duodenal lesions are normally associated with injury to other organs. The degree of duodenal trauma is classified according to the extension and the position of the lesion (Table 1). The incidence of associated duodenal injuries following trauma varies from 3 % to 5 %, but the incidence of isolated duodenal lesions is only about 0.6 % of patients with duodenal injury following abdominal trauma [5]. Road traffic accidents are the most common mode of blunt injury to the duodenum; other mechanisms frequently encountered in children are playground accidents, child abuse, falls, and bicycle handlebar trauma [6]. When injury occurs, the duodenum is generally compressed between the spine and external objects, such as the bicycle handlebar in our case. Presentation may be unspecific with abdominal pain and frequently with vomiting due to the haematoma compressing the duodenum, effectively creating a duodenal obstruction. Peritoneal signs and symptoms may present late, with general worsening of the patient’s overall condition [7]. A high white blood cell count is frequently found [7,8], while serum amylase levels may be elevated but less specific. Diagnosis may be delayed due to unspecific and sometimes subtle symptoms, strongly affecting the outcome [9]. Prompt diagnosis followed by correct and rapid treatment is crucial in this case; indeed a delay of more than 24 h can lead to a significant increase in morbidity and mortality. The mortality from small bowel injuries has been reported to be as high as 25.7 %, higher if diagnosis is delayed beyond 24 h [[9], [10], [11]]. Ultrasound is normally used in blunt abdominal trauma to give a rapid diagnosis, but may not be useful in the case of isolated duodenal trauma, as it mainly shows free intraperitoneal fluid which may be absent in such cases. The sensitivity for detecting intestinal injury has been reported as 34.7 % by sonography alone [12], while CT scanning has been reported to diagnose bowel injury in 88 % of blunt abdominal trauma cases. While contrast CT scan can easily identify the presence of free air in the retroperitoneum and abdominal peritoneum, and/or the presence of a periduodenal wall thickening with or without extravasation of contrast, exploratory laparotomy remains the definitive diagnostic test if a suspicion of duodenal perforation remains in absence of radiologic signs. Different surgical approaches have been described depending on the grade and type of lesion, but most lesions can be treated with a simple suture in single or double layer, with or without omental patch. In high-grade lesions, particularly in D2, due to the shared vascular supply with the head of the pancreas, the Roux-en-Y loop over the duodenal defect is the treatment of choice. In our case, the initial US performed at the Accident and Emergency department of our tertiary referral hospital was negative, but the persistence and rapid worsening of symptoms, particularly vomiting, prompted us to perform the IV contrast-enhanced CT scan that revealed the presence of free air in the retroperitoneal and peritoneal space, with an immediate suspicion of duodenal perforation. The prompt laparotomy revealed a duodenal haematoma with a laceration of the peritoneum and a perforation of the anterior part of the II duodenum. The distinctive feature of our case was the presence of a double isolated and asynchronous duodenal perforation after 4 days. The second perforation was in the first part of the duodenal bulb on the anterior wall, more than 1.5 cm proximally with respect to the previous one. While the possibility of a delayed intestinal rupture following blunt abdominal trauma is well-known, the difference in our case was the occurrence of two separate events at different times in two different locations of the duodenum, most probably due to traumatic compression of the common vascular supply of the I and II portion of the duodenum (superior pancreaticoduodenal artery), or a delayed extension of the intramural haematoma subsequent to the tangential blunt trauma. The initial surgical exploration showed no evident vascular damage in the location of the delayed perforation, apart from diffuse superficial suffusion.

**Table 1:** American Association for the Surgery of Trauma Organ injury Scale (AAST-OIS) for the duodenum [3].

GRADE^a^	Type of Injury	Description of injury
I	-Haematoma	-Involving a single portion of the duodenum
	- Laceration	- Partial thickness without perforation
II	-Haematoma	-Involving more than one portion of the duodenum
	- Laceration	- Disruption of < 50 % of the circumference
III	- Laceration	- Disruption of 50−75 % of the circumference of D2
		- Disruption of 50−100 % of the
		circumference of D1, D3, or d4
IV	-Laceration	- Disruption of > 75 % of the circumference of D2
		- Involving the ampulla or distal common bile duct
V	-Laceration	- Massive disruption of the duodenopancreatic complex
	-Vascular	- Devascularization of the duodenum

a Advance one grade for multiple injuries up to grade III. D1, D2, D3 and D4 indicate the first, second, third and fourth portions of the duodenum, respectively.

There are a number of points we would like to focus on. Despite the negative US, the CT scan permitted an immediate diagnosis, and the laparotomy was performed less than 7 h after the trauma. The careful positioning of the drain allowed safe management and monitoring of the post-operative period, and allowed us to detect the bile leakage from the second asynchronous perforation. To our knowledge, this is the first case reported in literature of an asynchronous double isolated duodenal perforation due to blunt abdominal trauma.

## 4 Conclusion

Isolated duodenal perforation due to blunt abdominal trauma is a rare event. In the event of blunt abdominal trauma with a negative ultrasound, the persistence or worsening of upper abdominal pain and the appearance of severe vomiting (triad: blunt abdominal trauma-upper gastric pain-vomiting), a contrast-enhanced CT scan is essential. Management of IDP following blunt abdominal trauma can be extremely challenging, but a prompt diagnosis is fundamental to avoid affecting the outcome. Although different surgical approaches have been described depending on the grade of lesion, it is our opinion that a single primary suture, possibly with an omental patch, should be the first choice of treatment, a position supported by the literature. Correct positioning of the drain is fundamental for post-operative monitoring. We suggest a scrupulous evaluation of the perfusion, of the anatomical distribution of the lesion and of the peripheral extension of the suffusion, when possible, to predict or suspect a subsequent perforation. To our knowledge, this is the first case reported in literature of an asynchronous double isolated duodenal perforation due to blunt abdominal trauma.

## Funding

**NO** funding or sponsor have been received.

## Ethical approval

**NO** ethical approval is needed for this **CASE REPORT**

## Consent

**"Written informed consent was obtained from the parents of the patient for publication of this case report and accompanying images. A copy of the written consent is available for review by the Editor-in-Chief of this journal on request”**.

## Author contribution

**Vito Briganti**: Project design, final revision, surgery

**Stefano Tursini**: Project design, final revision, surgery, figure selection, editing

**Stefania Ianniello**: Radiologic counseling, figure selection, revision

**Andrea Cortese**: Radiologic counseling, figure selection, revision

**Roberto Faggiani**: endoscopic counseling

## Registration of research studies

NO registration is due for this CASE REPORT

## Guarantor

**Guarantor: Dr. Stefano TURSINI M.D**.

## Provenance and peer review

Not commissioned, externally peer-reviewed

## Declaration of Competing Interest

The author declare **NO** financial and personal relationships with other people or organisations that could inappropriately influence the work. **NO** conflict of interest
